# Human iPSCs: atrial versus ventricular cardiomyocytes and their functional and pharmacological differences

**DOI:** 10.1042/BST20253028

**Published:** 2026-02-16

**Authors:** Steven David Broadbent, Jamie Ronald Bhagwan, Tosin Olusoga, Ashley Barnes

**Affiliations:** Axol Bioscience Ltd. Easter Bush, Edinburgh, EH25 9RG, U.K.

**Keywords:** Cardiomyocytes, Cardiovascular disease, Induced pluripotent stem cells, Pharmacology, Toxicology

## Abstract

The continuing development and characterisation of human-induced pluripotent stem cell (hiPSC)-derived cell-types has opened up a virtually endless source of human, physiologically relevant cells, available at scale, for scientific research. The technology’s maturation and refinement have allowed additional cell-types and sub-types to become available. The first step in adopting these novel cell-types is to properly characterise these cells and compare how they perform against the longer-established cell-types. Parallel to the progress in iPSC-derived cells has been the great strides in the platforms developed to assess and analyse the characteristics and functions of cells. These improved platforms have greatly increased the range, throughput and quality of the functional data that can be obtained from cell-types, including iPSC-derived cells. Research into cardiomyocytes in particular has been greatly enhanced by these platforms as cardiomyocytes not only have the expected cellular markers, proteomics and transcriptomics but are also electrically active and capable of contracting, opening a wide vista of potential assays. If human iPSC-derived cardiomyocytes are to confidently replace and supplement the existing animal and cellular models of the heart, it has to be demonstrated that they correctly replicate (or even improve) upon the functions and pharmacology of the existing heart models used on these new and improved platforms. Therefore, this review compares the functional and pharmacological differences seen between Axol’s human iPSC-derived atrial and ventricular cardiomyocyte cells on a range of established and newer platforms demonstrating the advantages of using chamber-specific human iPSC-derived cardiomyocytes and discussing how their use could supplement these emerging techniques.

## Introduction

The identification of the four ‘Yamanaka Factors’ in 2006, allowing the wide-scale production of human-induced pluripotent stem cells (hiPSCs) from primary tissue opened exciting new avenues in biological research [1]. For the first time, it was possible to create virtually endless amounts of human-relevant, primary-like cells consistently and at scale, without either the noted phenotypical and functional changes seen with immortalised cell lines (see [2] as an example) nor the potential ethical concerns around the use of embryonic stem cells. HiPSCs also present the opportunity to use a patient’s own cells for the creation of individualised clinically relevant models to more reliably predict the efficacy and toxicology of proposed treatment regimes for an era of truly-personalised precision medicine [3]. Further advances such as the development of the CRISPR gene-editing technique now offer the prospect of creating isogenic controls [3] and the march of ever higher-throughput assays and AI-facilitated ‘Big Data’ analysis is allowing the first attempts at cohort-level *in vitro* studies, dubbed ‘Clinical Trial in a Dish’ (CTIAD) [4].

Potential benefits of hiPSC-derived cells was rapidly recognised in the cardiovascular research space; with assessing the use of hiPSC-derived ventricular cardiomyocytes (vCMs) for more predictive cardiotoxicity models being an integral part of the large Comprehensive *in vitro* Proarrhythmia Assay (CiPA) Project set up in 2013 by the Health and Environmental Sciences Institute (HESI) [5], resulting in updated guidance on the clinical and non-clinical evaluation of pro-arrhythmia risk in 2022 [6]. The initial work supposedly assessed vCMs but the majority of commercially available and home-made hiPSC-derived cardiomyocytes (iCMs) are actually mixed populations of vCMs, and smaller sub-populations of atrial-like cardiomyocytes (aCMs) and sino-atrial node-like (SAN) cells [7]. Since then, protocols have been published producing purer populations of hiPSC-derived aCMs and SAN cells [8,9] and even chamber-specific vCMs such as a recent protocol producing pure left vCMs from hiPSCs [10].

One advantage of hiPSCs is that it allows iPSC-derived cells to be created from specific patients and family members, including those with rare mutations of unknown significance, which may otherwise be missed in population-based studies allowing the elucidation of the pathophysiological impact of these mutations [11]. Examples of the diseases that have been successfully modelled this way include Brugada Syndrome, Desmoplakin (DSP) Myocarditis and atrial fibrillation (AF) [11–13]. Furthermore as iCMs are amenable to CRISPR–Cas9 gene-editing, isogenic controls can be created and other genetic modification approaches used to tease out the subtleties of the effects of mutations of interest [12–14]. A number of cardiac diseases are also chamber-specific in their presentation such as AF and left ventricular hypertrophy (LVH) but also other diseases such as arrhythmogenic right ventricular cardiomyopathy and left ventricular DSP-associated cardiomyopathy being strongly associated with specific chambers of the heart, further underlining the need for chamber-specific CMs [13,15,16]. For instance, the Smith paper specifically cites the lack of chamber-specific Engineered Heart Tissue (EHT) as a drawback in their experimental model, one which easy access to hiPSC-derived aCMs and vCMs would help address [16].

These developments raised the question: do purer chamber-specific CMs provide any real benefit over simply using the standard mixed populations of CMs as produced by the established protocols and supplied by most commercial suppliers of human iCMs? To answer that, it is important to appreciate how a vCM differs in function, pathology, pharmacology and toxicology from aCMs. If large differences exist in baseline function and responses to pharmacological interventions, then this has significant impact on how effectively the iCMs can model the heart *in vitro*, especially when researching chamber-specific disorders such as AF and LVH.

AF in particular is of great interest given it represents the most common form of arrhythmia globally, affecting over 33 million people worldwide [17], a number which will continue to rise as the global population ages [18] along with a range of associated co-morbidities. Despite the pressing clinical need, there are only limited therapeutic options for treating AF with only poor success rates, 1- and 5-year mortality rates being 20% and 42.7%, respectively [19], Furthermore drug-induced AF is increasingly being recognised as an underappreciated risk factor in drug development [20] especially in cancer treatments [21]. To compound the clear clinical and toxicological needs for better AF models, many current AF treatments are known to have unexpected and deadly side-effects on vCMs potentially resulting in sudden cardiac failure [22] again underlining the need for better chamber-specific cardiac models.

## Proteomics and transcriptomics

**Figure 1:**
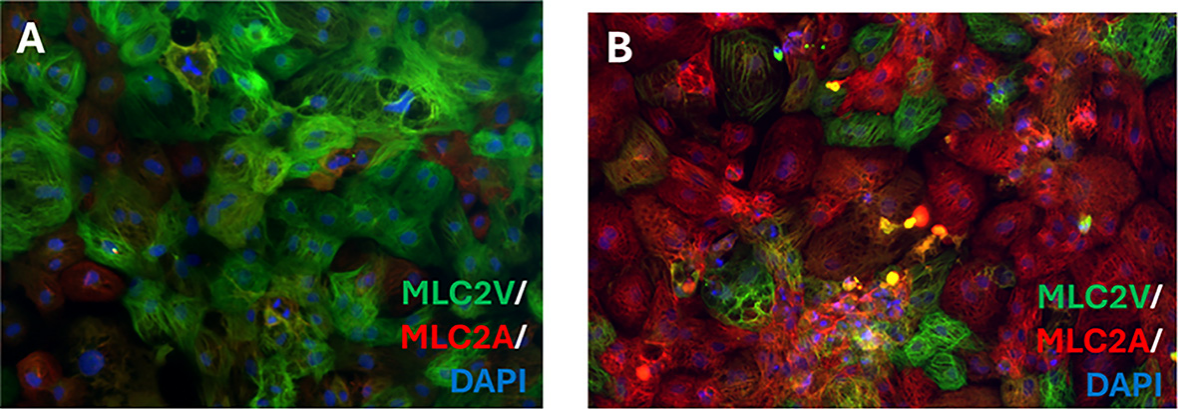
Chamber-specific iPSC-derived cardiomyocytes display appropriate chamber-specific immunocytochemistry markers. Representative immunocytochemistry imaging of hiPSC-derived cardiomyocytes for MLC2V (green), MLC2A (red) and DAPI (blue) from Axol’s isogenic hiPSC-derived vCMs (ax2508) (**A**) and hiPSC-derived atrial cardiomyocytes (ax2518) (**B**).

Differences in proteomics and transcriptomics of hiPSC-derived aCMs versus vCMs are discussed elsewhere in greater detail [23,24] and will not be covered in great depth here. However, the significant differences between the two will be acknowledged reflecting the differences seen between primary atrial and ventricular heart tissue, with for instance 77.8% of vCMs being found positive to the ventricular isoform of myosin light chain 2 and negative for the atrial isoform of myosin light chain 2 (MLC2V+/MLC2A–) and 94.7% of aCMs displaying the inverse MLC2V-/MLC2A+, ratios which remain fairly consistent across groups and commercial suppliers (for example [25]). For comparison, MLC2A expression in primary adult ventricular tissue is only around 21% of the level seen in primary adult atrial tissue [26]. These differences in iCMs can also be captured by the use of immunocytochemistry markers for MLC2A and MLC2V (Figure 1) which demonstrate appropriate chamber-specific marker staining in 80%+ of the cells. Similarly there are other key markers found in one chamber-specific cell-type and not the other with important impacts on cardiomyocyte function and pathophysiology. For instance, MYL4 encodes the atrial-specific essential light chain protein being replaced by MYL3 in vCMs as the heart develops [27,28]. Mutations in MYL4 cause a range of atrial dysfunction from arrhythmias and fibrillation through to fibrotic atrial cardiomyopathy and atrial standstill without affecting the corresponding ventricles [29]. This work was primarily performed in animal models and the potential of MYL4, for instance, as a novel therapeutic target requires replication in a more human model such as iCMs before attempting it in patients [30].

As also noted above, hiPSC-derived aCMs tended to be purer while hiPSC-derived vCMs were more of a mixed population. With these significant chamber-specific differences in transcriptomics and proteomics, one would predict corresponding differences in the functionality of the two cell types, in particular contractility, electrophysiology and pharmacology and it will be these differences this mini-review will focus on.

## Whole-cell patch-clamp (PC)

**Figure 2:**
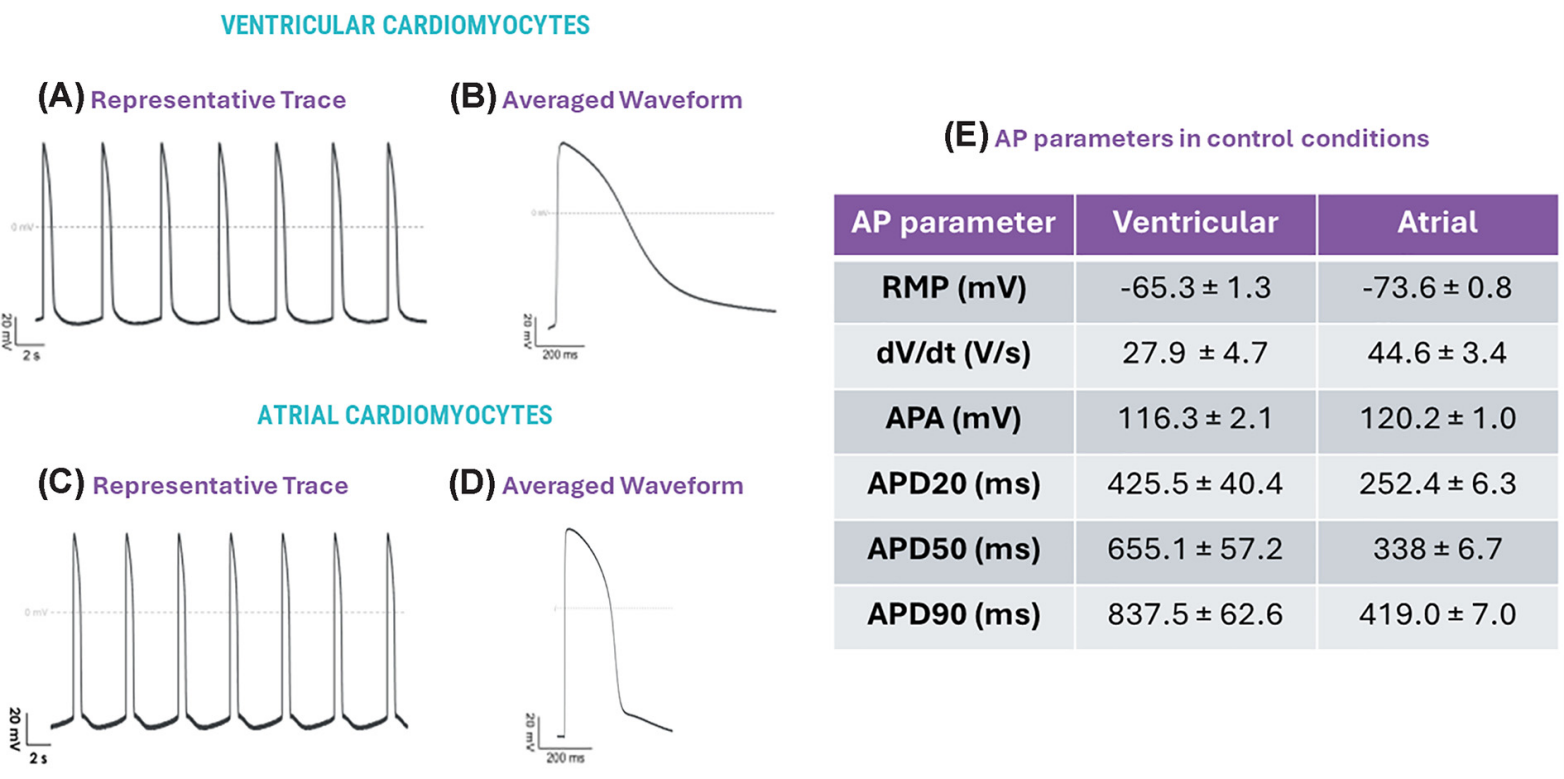
Chamber-specific iPSC-derived cardiomyocytes show distinct action potential waveforms. Representative traces (**A,C**), waveforms (**B,D**) and action potential characteristics (**E**) from spontaneous action potentials from Axol’s human iPSC-derived ventricular and atrial cardiomyocytes under control conditions using whole-cell patch clamp. RMP = Resting Membrane Potential, d*V*/d*t* = Slope, APA = Action Potential Amplitude APD_20/50/90_ = Action Potential Duration at 20%, 50% and 90% of repolarisation (data courtesy of Metrion Biosciences).

Whole-cell patch-clamp (PC) is considered the gold standard for assessing the functionality of electrically active cells such as cardiomyocytes, allowing the direct measurement of the electrical currents moving into and out of the cell. PC recordings from hiPSC-derived vCMs and aCMs (see Figure 2 for example) demonstrate that each cell-type produces characteristically different action potential waveform with vCMs producing longer waveforms with a slower upslope and more pronounced plateau phase while aCMs produce faster, shorter ‘more triangular waveforms’ findings replicated with other groups [24,31].

**Figure 3:**
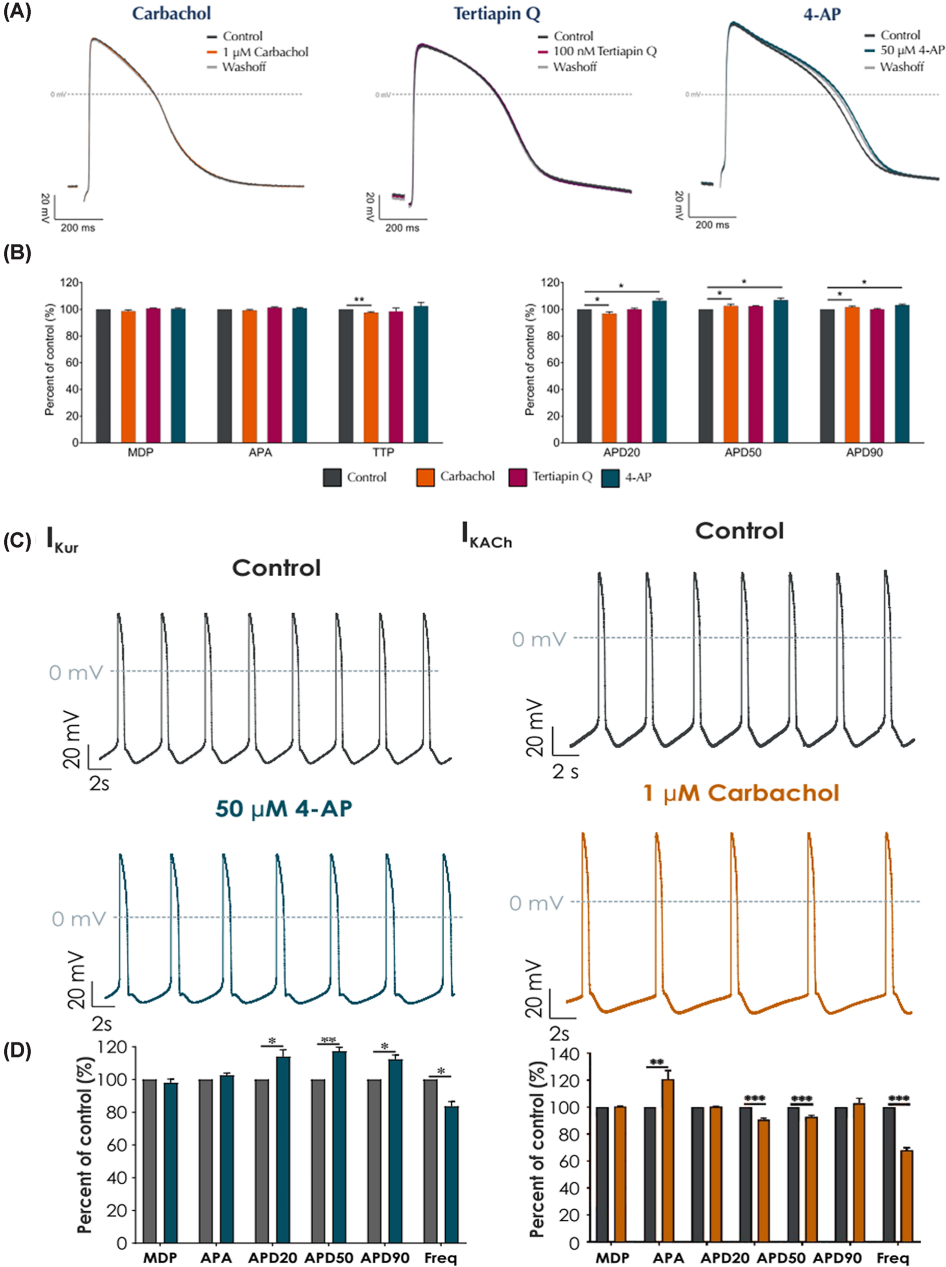
Chamber-specific iPSC-derived cardiomyocytes demonstrate distinct chamber-specific pharmacologies. Representative waveforms (**A**), action potential characteristics (**B,D**) and traces (**C**) from action potentials from Axol’s hiPSC-derived ventricular and atrial cardiomyocytes when treated with the atrial-specific compounds 4-AP, carbachol and tertiapin-Q (data courtesy of Metrion Biosciences).

Beyond differences in baseline functionality, differences in pharmacology can also be demonstrated using PC on vCMs and aCMs. A particular atrial-specific current of interest is the *I*_K,ACh_ current. The literature shows that effects on the atrial-specific *I*_K,ACh_ current by carbachol and adenosine can be detected in hiPSC-derived aCMs by PC while being completely absent in hiPSC-derived vCMs [25]. The presence of these atrial-specific currents in aCMs, but not vCMs, has been confirmed with commercially-available chamber-specific iCMs. Figure 3 compares the action potential waveforms and characteristics of vCMs and aCMs in the presence of atrial-specific compounds such as 4-AP, carbachol and tertiapin-Q. The vCM action potential waveform (Figure 3A) and characteristics (Figure 3B) show minimal effects to 1 μM carbachol, 50 μM 4-AP and 100 nM tertiapin-Q underlining the ventricular-like pharmacology of human iPSC-derived vCMs. In contrast, carbachol and 4AP produce significant changes in beat rate (BR, Figure 3C), action potential waveforms and characteristics (Figure 3D) and while 4-AP significantly increased the action potential duration (APD), carbachol decreased it, correctly reproducing their atrial-specific pharmacologies. The clear differences in chamber-specific pharmacologies demonstrates the benefit of using chamber-specific iCMs when assessing compound efficacy and toxicology.

## Multi-electrode array (MEA)

**Figure 4:**
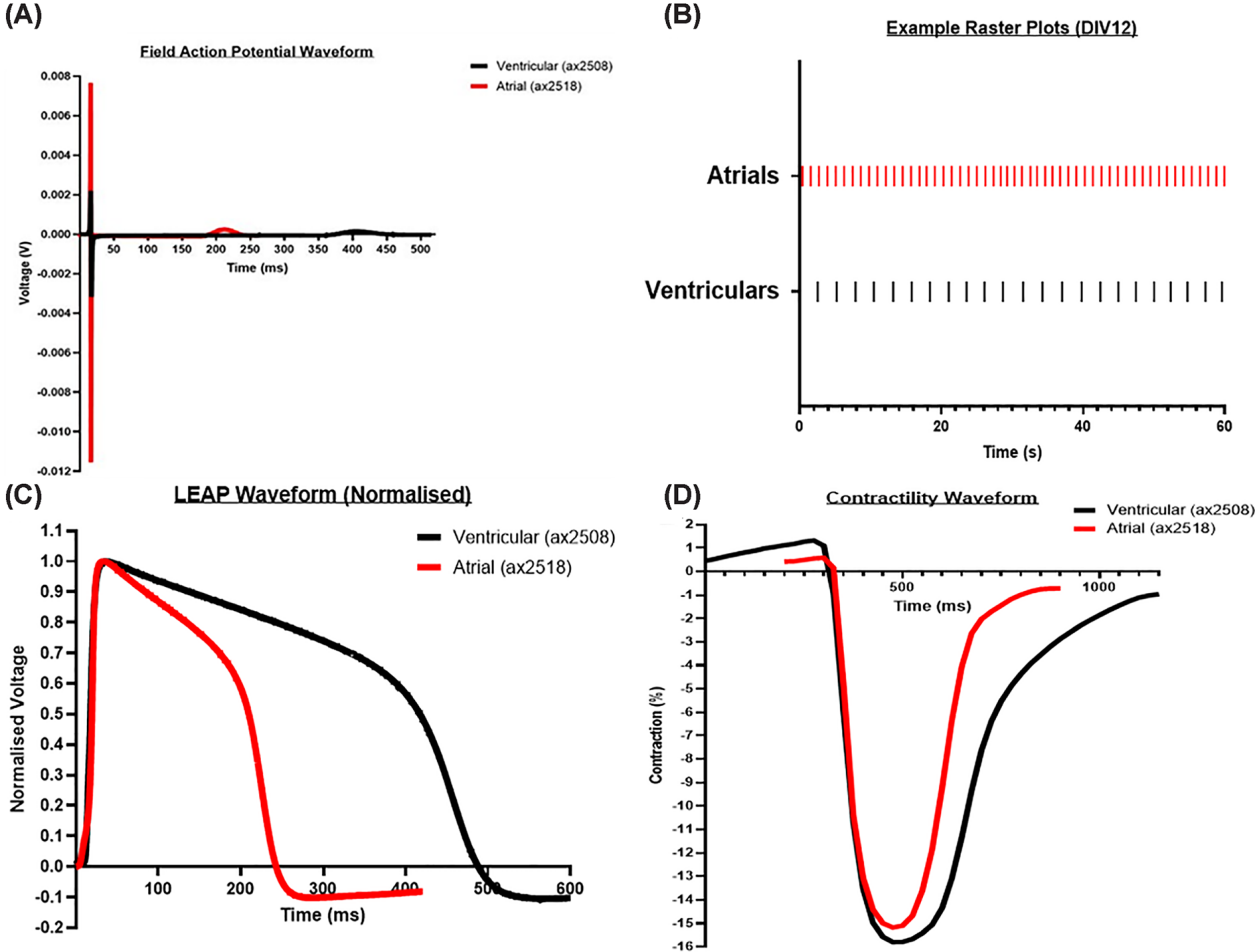
Chamber-specific iPSC-derived cardiomyocytes show distinct beat rates, fAP waveforms, contractility waveforms and LEAP waveforms on MEA platforms. Representative fAP traces (**A**), raster plots (**B**), LEAP waveforms (**C**) and contractility waveforms (**D**) from Axol's human iPSC-derived ventricular and atrial cardiomyocytes under control conditions using the Axion MEA system (Axol Bioscience in-house data).

The chamber-specific differences in the electrophysiology of hiPSC-derived aCMs and vCMs can also be demonstrated on other platforms such as multi-electrode array (MEA). MEA systems offer a higher throughput alternative to the PC technique with a range of different modalities, especially when used with cardiomyocytes. The Axion MEA system (Axion Biosystems Inc., U.S.A.), for example, not only offers the standard MEA field action potential (fAP) option but also an impedance-based contractility modality and the PC-like local extracellular action potential (LEAP) modality in formats up to 384wp. This flexibility and throughput saw MEA systems widely-utilised with hiPSC-derived vCMs in the extensive CiPA study [5]. These advantages can also be applied to examining differences in chamber-specific iCMs. Figure 4 shows some representative baseline data obtained from Axol’s aCMs and vCMs. Figure 4A shows a typical cardiomyocyte fAP from aCMs and vCMs. As can be seen, the aCMs have a significantly shorter Field Potential Duration (equivalent to the Patch Clamp APD_90_ value) mirroring the findings obtained using the PC method (Figure 2). The Raster Plot (Figure 4B) also replicates findings in the literature that hiPSC-derived aCMs have faster BRs than vCMs (see [32] as an example). The LEAP data in Figure 4C demonstrate the same relative waveform shapes and duration as seen with PC; with aCMs being more triangular with a shorter APD while vCMs are longer-lasting with a pronounced calcium plateau which was also replicated with the contractility module (Figure 4D).

## Contractility

Although the contractility module is a useful add-on to the Axion’s central fAP function, because it assesses contractility via impedance, it can only indirectly measure contractility and can therefore only provide relative changes. In contrast, dedicated contractility platforms allow the direct measurement of the force of contraction such as the innoVitro FLEXcyte 96 (innoVitro GmbH, Germany), a specially-designed plate which works on the Nanion CardioExcyte 96 platform (Nanion Technologies, Germany). Briefly the FLEXcyte 96 uses a high-precision, ultra-thin, hyperelastic polydimethylsiloxane (PDMS) membrane with a physiologically relevant elastic modulus of 30 kPa, to recapitulate the native cardiac environment to which the iCMs can directly adhere. As the cells beat, this membrane flexes and the amount of mechanical stress produced can be measured allowing the direct measurement of the amount of force the syncytium produces. Axol has worked very closely with innoVitro to characterize our aCMs and vCMs, assessing both their baseline activity and compound effects. These data are covered in more detail in a joint innoVitro/Axol publication [33] but a brief overview will be presented here.

**Figure 5:**
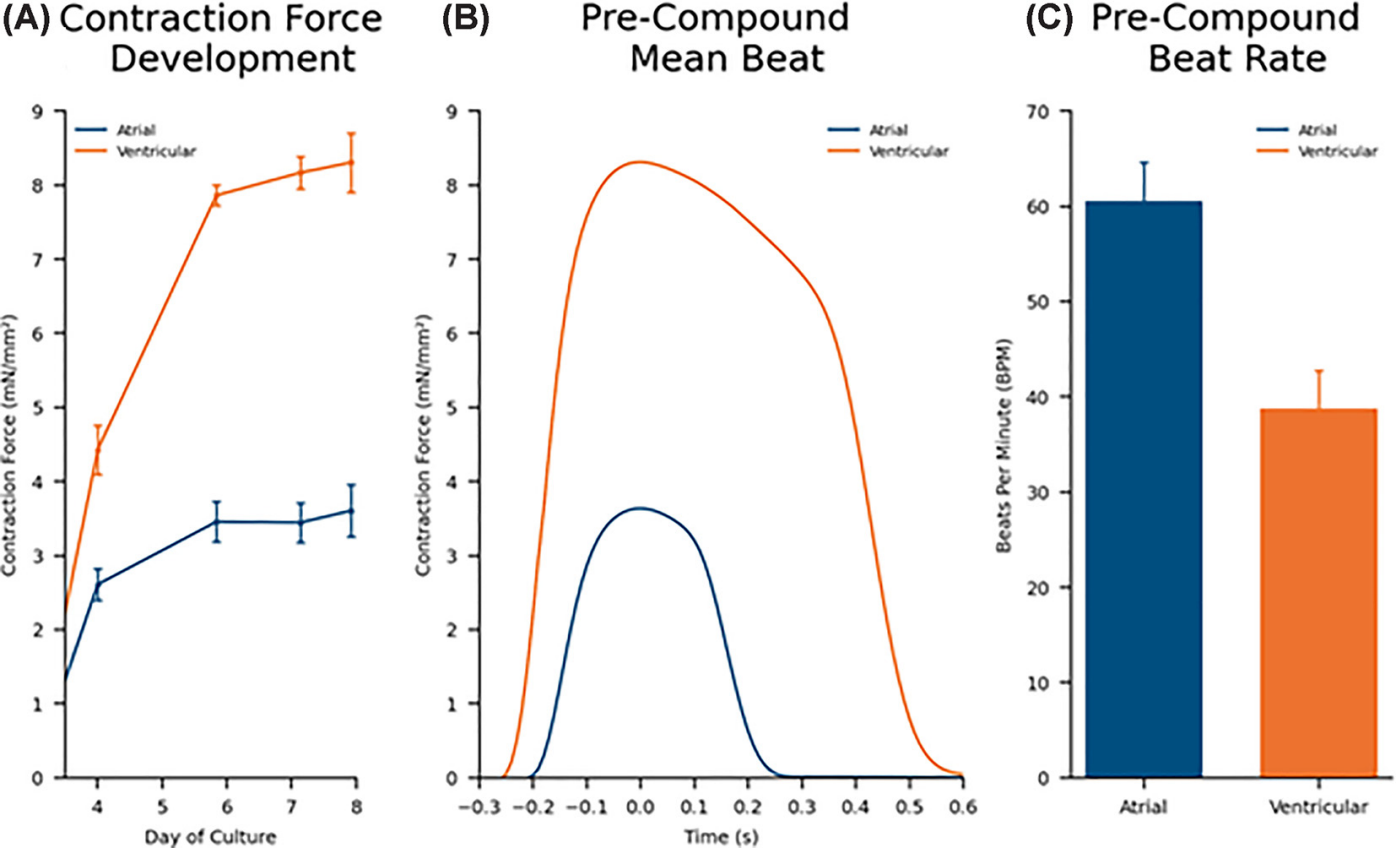
Chamber-specific iPSC-derived cardimomyocytes produce different amounts of contractile force and distinct contractility waveforms on the FLEXcyte 96 contractility platform. Comparison of differences in Contraction Force Development (**A**), the Pre-Compound Mean Beat Waveform (**B**) and Pre-Compound Beat Rate (**C**) of Axol's human iPSC-derived atrial and vCMs as recorded using the FLEXcyte 96 contractility platform (data from Lickiss et al. 2024).

Data from the FLEXcyte platform (Figure 5) replicates the general observation of baseline activity of Axol’s aCMs and vCMs using PC and the different modalities of the MEA system, namely that aCMs contractions are shorter (Figure 5B) with a higher BR compared with the corresponding vCMs, with vCMs having a more pronounced plateau phase. Additionally, the direct measurement of force using the FLEXcyte 96 supplements the MEA’s impedance-based indirect contractility measurement to demonstrate that vCMs beat with greater force than the corresponding isogenic aCMs (Figure 5A).

**Figure 6:**
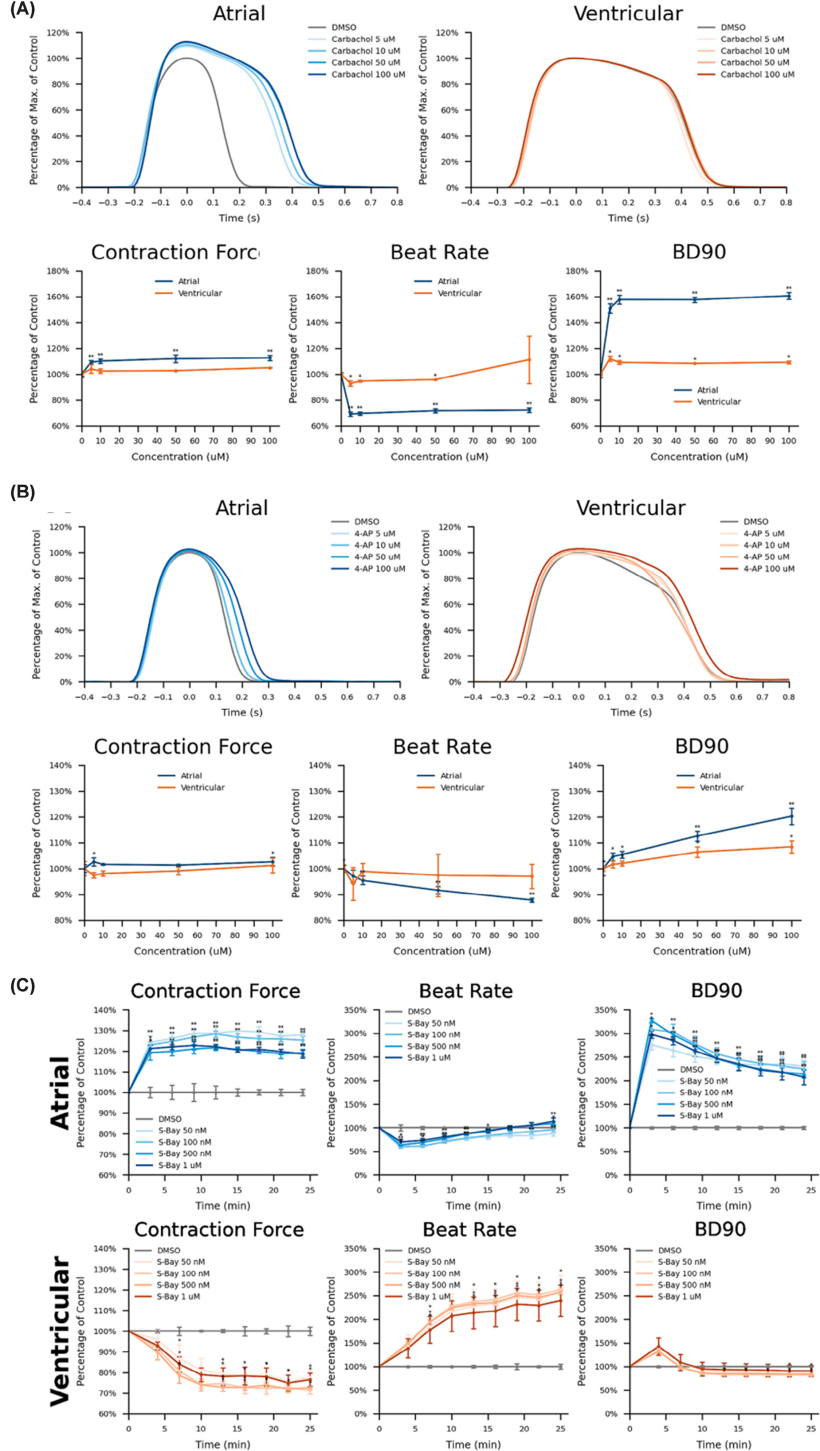
Chamber-specific iPSC-derived cardiomyocytes have distinct pharmacologies as assessed on the FLEXcyte 96 contractility platform. Comparison of compound effects on Axol’s human iPSC-derived atrial and vCMs' contractility demonstrating effects on waveform, contraction force, beat rate and Beat Duration90 when treated with carbachol (**A**), 4-AP (**B**) and S-Bay K8644 (**C**) (data from Lickiss et al. 2024)

Having established different baseline contractility activities for the aCMs and vCMs (Figure 5),these differences are only further amplified by the addition of compounds (Figure 6). The atrial-specific acetylcholine receptor agonist carbachol, for instance, significantly extends the beat duration (BD) of the aCMs, and increases contractile force (CF) while markedly reducing the BR (Figure 6A). These results replicate the effects produced in aCMs using whole-cell PC (Figure 3A,B) and the wider literature where carbachol is reported to have a biphasic inotropic effect; an initial negative inotropic effect followed by a positive inotropic effect appearing at higher concentrations or after time, in mouse and human atrium, thought to be mediated by muscarinic receptors [34–37].

In contrast, even the highest concentrations of carbachol had no noticeable effect on the vCMs waveform or CF and only minor effects on BR and BD (Figure 3C,D). The atrial-specific 4-AP (Figure 6B) only produced modest changes to the Contractility waveforms and CF of both aCMs and vCMs compared with carbachol but markedly different effects on BR and BD. with the aCMs being significantly more affected, also replicating the whole-cell PC data (Figure 3). These subtleties of compound effects, both within the same platform and between platforms, highlight the importance of looking at a range of parameters and multiple platforms to get the most accurate picture of the cardiac effects of compounds.

Most interestingly are the divergent effects of S-Bay K8644, an L-type calcium channel agonist on aCMs and vCMs (Figure 6C). While it could be predicted that S-Bay K8644 would be generally cardioactive, it would not be inherently obvious that its effects would show marked chamber-specificity. However, the data show that while S-Bay K8644 is a positive inotrope on aCMs, it produces negative inotropic effects on vCMs. Similarly, S-Bay K8644 initially reduces the BR on aCMs while markedly increasing it in vCMs and while BD is markedly and chronically increased in aCMs, it’s only transiently and moderately affected in vCMs. This unpredicted chamber-specific effect illustrates the utility of using chamber-specific, donor-matched samples for *in vitro* drug screening assays to flag up such unexpected responses. Had S-Bay K8644 been a completely novel compound, undergoing initial cardiac-efficacy and cardiotoxicity testing, then different initial conclusions could have been drawn depending on the chamber-specific iCMs used, with significant ramifications on subsequent *in vivo* testing.

## High-throughput automated patch clamp

Despite the impressive developments in MEA and contractility platforms, whole-cell PC remains the gold standard for assessing the pharmacology and ion channel function of cardiac cells, especially for cardiotoxicity [6]. However, manual PC remains limited by its throughput and difficulty, so extensive efforts have been made to develop higher-throughput automated patch clamp (APC) systems. To this end the two biggest players in this field, Nanion and Sophion, have separately developed and validated APC systems for cardiomyocytes with Nanion developing the Synchropatch 384 and Sophion the Qube 384. As the names indicate these systems attempt to patch 384 cells in parallel and even if most cells fail to be successfully patched (publications indicate an overall 20–30% success rate in patching native or iCMs), this still represents orders of magnitude improvements in throughput compared with manual PC [38]. As the technology has been refined and protocols optimised reported success rates have improved with 40% and 75%+ success rates reported in the literature and conference communications [39,40]. These improvements have seen APC systems hailed as the new gold standard for the pharmaceutical industry at least [39]. The initial high entry price has restricted take-up of APCs by academics however the technology is slowly being adopted with both APC platforms being used in academic publications in recent years demonstrating its arrival as a viable technology (e.g [41,42]).

## 3D cultures, organ-on-a-chip and microphysiological systems

The ability to grow and study cells in 2D monolayers cultured on glass or plastic surfaces has been immensely beneficial in terms of ease of access, simplicity and throughput. However, such a culture remains distinctly unphysiological due to the immature phenotype exhibited by cardiac cells in 2D. An increasing body of evidence highlights the importance of the extracellular matrix (ECM) on which the cells are cultured for their development and function, as well as the reality that biological processes occur in 3D [43,44]. Whether in a heart, a brain or any other organ, cells typically develop and function as part of a larger 3D structure and this has impact on the individual cells and has important ramifications on the temporal and spatial organisation and function of the larger organ, tissue and the organism as a whole [45]. For instance, the aCMs and vCMs not only differ individually in terms of pharmacology, protein expression, BR, APD etc. as discussed previously but, *in vivo*, they are also physically separated into anatomically distinct chambers and during the heartbeat, contract and relax in a strict temporal order. To better capture this level of complexity, innovations have been introduced into how the cells are cultured, moving away from an unphysiological monolayer to something better resembling *in vivo* conditions.

The simplest development is to build on the work by Sato *et al.* and grow cells in 3D organoids [46]. At its most basic the seeding and culture conditions allow the growing of one or more cell-types in simple 3D structures, typically a sphere, either from a stem cell, progenitor cell or a more differentiated cell-type; the ‘cardiospheres’ resulting from this process have been developed and validated by multiple groups [47]. Even the introduction of this basic 3D organisation has been reported to produce functional benefits and the development of a more complex stratified structure. Cardiospheres have been demonstrated to exhibit multilineage differentiation with the cells self-organising into distinct layers, with proliferating cardiac progenitors located in the core of the cardiosphere while the peripheral cells, representing a mix of endothelial, mesenchymal and/or cardiomyogenic lineages, provide support to the progenitors [47]. The cardiac stem cells present in turn can release their own local morphogens, driving greater levels of structure organisation developing relevant rudimentary anatomical characteristics such as chamber organisation and distinct atrioventricular specifications [48].

While cardiospheres generally self-assemble, 3D-cultures can also undergo directed assembly by the operator allowing guidance of the final structure [44]. Such cultures are referred to as organoids, assembloids or EHTs according to differences in underlying technique, although authors will often use them interchangeably with no hard and fast agreed distinctions and often experimenters combine techniques from multiple approaches to optimise the end result therefore, for convenience, we shall use organoid as the general catch-all term [44]. Regardless of precise description all organoids employ a combination of techniques to better direct the final 3D structure of the culture and improve the physiological relevance of their model. These techniques include co-culture with other cell-types, choice of ECM, specially-designed microwell moulds, biocompatible pillars, 3D-printed scaffolds and custom-designed bioreactors [44]. In the first instance these different forms of 3D culture provide improved, more-physiological cardiac models compared to their 2D counterparts which, combined with specific donor-derived iPSCs, also offers the potential of specific patient organoids for personalised medicine and even a possible source of transplant material, bypassing the ethical, logistical and immunological challenges of using donor tissue [49].

These clever culturing techniques generate layered cultures and rudimentary anatomical structures within a single organoid; however, the use of microphysiological systems (MPS) has allowed cultures to be further engineered in an approach known as organ-on-a-chip (OOC). These OOC systems use cell culture chips engineered with separate chambers to allow the culture in 2D and 3D of monolayers and/or organoids of specific cell types connected by channels to replicate physiological fluid flows and the innervation by neurites, while allowing parallel seeding and differentiation of different cell types better modelling organ-level physiology [50]. Such OOC systems can also allow drug additions to be carried out separately on different organ systems and the incorporation of microelectrodes allows the measurement of electrical activity and/or contractility [51]. As an example, an OOC system might include a gut-cell chamber connected to a liver-cell chamber connected to a heart-cell chamber thereby mimicking the journey of an orally-taken drug passing (or not) the gut-barrier, then first-pass metabolism by the liver, with the heart cell only being exposed to whatever remains of original unmetabolised drug and any active metabolites, in a way currently impossible outside of an *in vivo* study. The precise makeup and complexity of the model can then be specifically tailored as we move towards so-called body-on-a-chip models, with up to eighteen-organ MPS allowing the modelling of the full ADME journey [52].

The spectrum of options from basic 2D monolayers through cardiospheres, organoids, assembloids and EHTs to the most complex multi-organ OOCs has set-up a fascinating dynamic. Each step along that chain increases the physiological relevance of the model but at the expense of simplicity of use and throughput and vice versa. Therefore groups working on monolayers and organoids continually seek to improve the physiological relevance of their model while the groups working on OOC platforms seek to improve throughput and ease of use. Techniques are being used to improve the functional relevance of the simpler cell models including the use of co-culture, optimising ECMs, electrical stimulation protocols and maturation media (for a review see [53]) while both academic and commercial groups report scaling their MPS systems to allow up to 16 separate assays per chip, with more to be added until some unsurpassable physical limitation is reached [54,55] meaning the true sweet spot between throughput and physiological relevance remains to be determined.

## ‘Big Data’ and AI

The developments discussed above along with the development and refinement of other techniques present their own challenges to traditional manual data-processing and analysis practices. Safety and efficacy studies can produce immense amounts of data from the likes of APC and High Content Imaging and the ever-growing array of *‘omics* (genomics, transcriptomics, proteomics, epigenomics, metabolomics, etc.) which presents both challenge and opportunity for data interpretation [56].

This is compounded by the push to identify potential concerns around putative treatments’ effectiveness and safety earlier in the drug development pipeline. For example, identifying potential cardiotoxic liabilities during early-stage testing on cell lines rather than during animal studies, clinical testing or post-marketing surveillance represents savings of millions of dollars in development cost and months/years of wasted effort and the potential avoidance of harm when the drug goes into humans [6]. The challenge therefore becomes that rather than assessing a handful of lead compounds, panels of hundreds or even thousands of compounds are tested, sometimes over multiple concentrations. The potential of ‘Big Data’ to either “*empower or overwhelm*’ has been long known as has the need to appropriately handle the challenge of ‘Big Data’s’ 5Vs (‘*volume, veracity, velocity, variety and value*’) [57].

iPSC-derived cells are not immune from these concerns and have the potential to exponentially increase the volume of data available both in terms of the inherent complexity of, say, iCMs compared with hERG-expressing HEK cells, but also from multi-donor CTIAD approaches and the innovative ‘Village in a Dish’ adaptation where results from hundreds, even thousands, of unique individuals need to be processed [4,58].

Faced with this deluge of data the obvious solution is to lean into the advancements in AI as it is only through automating data curation, handling, processing and analysis that meaningful conclusions from such immense and varied datasets can ever be drawn. Therefore groups are actively developing AI tools to automate the analysis of large-scale datasets obtained from iPSC-derived cells. For example, machine learning models have been shown to be able to correctly identify seizure and neurotoxicity liabilities with an accuracy of 99.9% from MEA data recorded from hiPSC-derived neuronal cells [59] with similar efforts being made to use AI with iCMs and the first AI-developed therapeutics [50].

This relationship between iPSC-derived cell models and AI is increasingly driving a virtuous development circle where data from iPSC-derived cells are used to initially train the AI model, which is then used to virtually design and screen novel therapeutics, producing a much-shorter list of candidates to be assayed against iPSC-derived cells to provide validation of their safety and/or effectiveness greatly streamlining and accelerating the development process, which in turn provides additional data for future training [50]. This process of iterative augmentation will only continue to improve and deepen this relationship between AI and iPSC-derived cellular models [60].

## Conclusion

In conclusion this review has examined how the development of purer chamber-specific human iCMs provides real experimental benefit over using standard mixed populations of iCMs and how the differences in baseline activity, compound effect and cardiotoxicity can be captured using a number of different platforms. The stark chamber-specific differences seen in both the baseline and compound-treated functions of hiPSC-derived aCMs and vCMs, demonstrates the benefit of their use both generally and with clear chamber-specific disorders such as AF or LVH. These conclusions also support developing additional hiPSC-derived cardiac subtypes such as left ventricular and SAN cardiomyocytes [9,10]. Finally, we've explored how using these chamber-specific iCMs could augment the recent developments in assays and analysis such as APC and AI and the potential synergy of these emerging technologies offers great potential for future cardiac research.

## Perspectives

- Human iPSC-derived cells offer the potential of a large-scale source of human, physiologically relevant cellular material for research; however, widespread adoption requires the demonstration of the utility of the different cell-type and sub-cell-type specific forms of human iPSC-derived cells and their functional relevance.
- As part of this, there is a proven need for better, more chamber-specific hiPSC-derived cardiomyocytes to meet the currently unmet challenges of cardiac conditions such as atrial fibrillation. The growth and improvement in the range of platforms available has also increased the range of characteristics that can be examined in cells such as hiPSC-derived cardiomyocytes, allowing a broader picture of the functional and pharmacological differences of different cell-types.
- The platforms and techniques reviewed in this communication can be used to better characterise hiPSC-derived atrial and vCMs; both healthy and diseased and can be extended to future novel hiPSC-derived cardiac cell-types such as left ventricles and sino-atrial nodal cells. Similar approaches can be used to better characterise other hiPSC-derived cell types such as neuronal and glial cells.

## Data Availability

As a mini-review, only the data in Figure 4 are original data curated by Axol. Should a researcher wish to access the data displayed in Figure 4, they are encouraged to contact the corresponding author to facilitate access.

## Open Access

Open access for this article is provided via our Subscribe to Open programme, made possible through the support of our subscribing institutions. Learn more here: https://portlandpress.com/pages/open_access_options_and_prices#conditional.

## Abbreviations

APD: action potential duration
aCM: atrial cardiomyocyte
AF: atrial fibrillation
APC: automated patch clamp
BD: beat duration
BR: beat rate
CTIAD: clinical trial in a dish
CiPA: Comprehensive *in vitro* Proarrhythmia Assay
CF: contractile force
DSP: Desmoplakin
EHT: Engineered Heart Tissue
ECM: extracellular matrix
fAP: field action potential
HESI: Health and Environmental Sciences Institute
hiPSC: human-induced pluripotent stem cell
iCM: iPSC-derived cardiomyocyte
LVH: left ventricular hypertrophy
LEAP: local extracellular action potential
MPS: microphysiological systems
MEA: multi-electrode array
OOC: organ-on-a-chip
PC: patch-clamp
PDMS: polydimethylsiloxane
SAN: sino-atrial node
vCM: ventricular cardiomyocyte
